# 60 Childhood Behçet disease: clinical features, management and outcome of 13 cases

**DOI:** 10.1093/rheumatology/keac496.056

**Published:** 2022-10-07

**Authors:** R El Qadiry, H Nassih, A Bourrahouat, I Ait Sab

**Affiliations:** Pediatric B Department—Mother-Child Pole—Mohammed VI University Hospital, Marrakesh

## Abstract

**Background:**

Behçet disease is a rare vasculitis that involves medium and small calibre arteries. Multisystem involvement is possible. Few are the papers that provide information about its clinical course, and outcome in the pediatric population. Also, the management is mainly based on adult guidelines.

**Objectives:**

To shed light on the clinical course and outcome of juvenile Behçet disease.

**Methods:**

A single center retrospective descriptive study of 13 children with Bechet disease. The mean follow-up period was for 3 years.

**Results:**

We had 8 boys and 5 girls. Four of our patients were consanguineous. The mean age was 9 years. The mean interval between onset and diagnosis was 3 months. All of our patients had recurrent aphthous stomatitis with a median of 6 episodes per year. The other symptoms at diagnosis were concomitant genital ulcers in 4 cases, pseudo-folliculitis in 7 cases, urticarial rush in 4 cases, severe headache in 2 patients, stroke in one patient, prolonged and/or recurrent fever in 9 patients, uveitis in 4 cases, episcleritis in one case, arthritis in 5 cases, pulmonary hemorrhage in one case, and pyoderma gangrenosum in one case. The pathergy test was performed in all the patients, and was positive in 3 of them. Diagnosis was based on the International Criteria for Behcet's Disease. Acute phase reactants (ESR and CRP) were high in all patients. Meanwhile, granulocytosis was found in 10 patients. HLA 51 was positive in 8 patients, from whom three were siblings. All our patients were on steroids, five had colchicine, seven had azathioprine, two had methotrexate, two had cyclophosphamide, three had etanercept and one had tocilizumab. Complete remission was obtained in 8 cases in a mean time of 15 months, while five children had partial remission, and one boy lost sight of his left eye after a severe retinitis.

**Conclusion:**

Behcet’s disease can cause severe sequalae if not diagnosed and treated on time. More studies are needed to establish management guidelines for pediatric population.

